# mPEG@ELA-11 Alleviates Atherosclerosis via AKT-ER Stress-Mediated Macrophage Modulation

**DOI:** 10.34133/bmef.0203

**Published:** 2025-11-25

**Authors:** Xiaoguang Li, Ning Dou, Linshan Zhong, Yicheng Wu, ZhenZhen Cai, Zaixu Zhao, Lefeng Qu, Qixia Jiang

**Affiliations:** ^1^ Center of Vascular Diseases, Shanghai Fourth People’s Hospital Affiliated to Tongji University School of Medicine, Shanghai 200434, China.; ^2^ Department of Vascular and Endovascular Surgery, Changzheng Hospital Affiliated to the Naval Medical University, Shanghai 200003, China.; ^3^ Department of Cardiovascular Medicine, Tongren Hospital, Shanghai Jiao Tong University School of Medicine, Shanghai 200336, China.; ^4^ Department of Clinical Laboratory, Shanghai Fourth People’s Hospital Affiliated to Tongji University School of Medicine, Shanghai 200434, China.; ^5^ Department of Biological Medicines and Shanghai Engineering Research Center of Immunotherapeutics, School of Pharmacy, Fudan University, Shanghai, China.; ^6^ Department of Cardiology, Shanghai General Hospital, Shanghai Jiao Tong University School of Medicine, Shanghai 200080, China.

## Abstract

**Objective:** This study explores the role of methoxy polyethylene glycol@Elabela-11 (mPEG@ELA-11), a pH-responsive ELA-11 conjugate, in modulating macrophage function and attenuating atherosclerosis, focusing on the protein kinase B (AKT)-mediated endoplasmic reticulum (ER) stress pathway as a molecular target. **Impact Statement:** We reveal that ELA-11 alleviates atherosclerosis by suppressing macrophage foam cell formation, M1 polarization, and apoptosis via the AKT-ER stress pathway. We also develop mPEG@ELA-11, a novel pH-responsive nanocarrier, to enhance targeted drug delivery and therapeutic efficacy, offering a breakthrough for peptide-based cardiovascular nanomedicine. **Introduction:** Atherosclerosis, driven by macrophage dysfunction and lipid accumulation, is a major global killer. ELA-11, a fragment of Elabela peptide, shows cardiovascular protective effects, but its role in atherosclerosis and optimal delivery remain unstudied. **Methods:** Elabela mRNA (APELA) expression was analyzed in human carotid atherosclerotic plaques using real-time quantitative PCR analysis, and serum ELA levels were quantified via enzyme-linked immunosorbent assay in patients with carotid stenosis. In vitro studies on RAW264.7 macrophages evaluated mPEG@ELA-11 effects on oxidized low-density lipoprotein-induced foam cell formation, polarization, and apoptosis. In vivo efficacy was tested in ApoE^-/-^ mice, comparing mPEG@ELA-11 with free ELA-11, and its pH-responsive release mechanism was characterized. **Results:** APELA was down-regulated in human atherosclerotic plaques, especially unstable lesions. mPEG@ELA-11 suppressed foam cell formation, M1 polarization, and apoptosis by inhibiting the AKT-ER stress pathway in vitro. In mice, it reduced plaque area more effectively than free ELA-11 attributed to pH-triggered release. **Conclusion:** The pH-responsive mPEG@ELA-11 alleviates atherosclerosis by modulating macrophages via the AKT-ER stress pathway, with favorable targeting and safety, representing a promising targeted peptide nanomedicine for atherosclerosis.

## Introduction

Atherosclerosis (AS) is a multifaceted chronic inflammatory disorder characterized by the accumulation of lipids and inflammatory cells within the arterial wall. It is the primary cause of both cardiovascular and peripheral arterial diseases, posing a substantial global health burden [1,2]. In 2020, approximately 19 million individuals succumbed to cardiovascular diseases worldwide, marking an 18.7% increase from 2010 [3]. Since 2000, ischemic heart disease has emerged as the leading contributor to this mortality, accounting for 16% of all global deaths [4].

The pathophysiology of AS involves a complex interaction between various cell types, with macrophages playing a pivotal role [4]. Under normal physiological conditions, high-density lipoprotein (HDL) prevents cholesterol accumulation in macrophages. However, risk factors such as hypercholesterolemia and hypertension facilitate the retention of modified lipoproteins in the subendothelial space [5]. Macrophages subsequently engulf these modified lipoproteins, particularly oxidized low-density lipoprotein (ox-LDL), and transform into foam cells. This process, coupled with macrophage apoptosis, contributes to plaque formation and progression [6].

Macrophages within atherosclerotic lesions exhibit phenotypic plasticity, polarizing into pro-inflammatory M1 or anti-inflammatory M2 subtypes [7]. M1 macrophages, activated by interferon-γ and lipopolysaccharide, produce pro-inflammatory cytokines that exacerbate atherosclerotic progression. In contrast, M2 macrophages, induced by interleukin-4 and interleukin-13, secrete anti-inflammatory factors that mitigate plaque development. Thus, modulating macrophage polarization presents a promising therapeutic avenue.

Recent research has increasingly focused on peptide-based strategies for preventing and treating AS. Peptide therapeutics offer advantages including high specificity, potent bioactivity, and minimal side effects. For example, glucagon-like peptide-1 receptor agonists (GLP-1RAs) have demonstrated potent anti-atherosclerotic effects in animal models [8] and have been shown to reduce the incidence of major adverse cardiovascular events in clinical settings [9].

Elabela/Toddler (ELA) is an identified endogenous peptide that exerts its effects through the G protein-coupled receptor angiotensin receptor-like 1 (APJ) [10]. ELA is expressed in various tissues, including the vascular endothelium, and plays critical roles in both embryonic development and cardiovascular function [11]. The mature ELA-32 peptide is cleaved into smaller bioactive fragments, including ELA-21 and ELA-11 [5].

Studies have highlighted the importance of ELA and its receptor system in cardiovascular pathologies. ELA deficiency or APJ knockout in mice leads to severe AS and vascular structural abnormalities [12]. Although Apelin, another APJ ligand, has demonstrated anti-atherosclerotic effects, its short half-life (5 to 8 min) limits its therapeutic potential [13]. In contrast, ELA-32 exhibits a longer half-life of 47.2 ± 5.7 min [14], positioning it as a more viable candidate for therapeutic development.

Among ELA fragments, ELA-11 stands out due to its short peptide chain and high lipophilicity, which enable it to effectively activate the APJ receptor and its downstream signaling pathways [15]. However, the precise role of ELA-11 in AS and its impact on macrophage function remain to be fully elucidated.

Endoplasmic reticulum (ER) stress has emerged as a critical factor in the pathogenesis of AS [16]. ER stress serves both as a cause and consequence of atherosclerotic development [17]. Chronic ER stress can lead to macrophage apoptosis and plaque necrosis, contributing to plaque instability [18]. The unfolded protein response (UPR), activated by ER stress, involves 3 primary pathways mediated by IRE1, ATF6, and PERK [19]. Notably, the PERK pathway has been implicated in macrophage apoptosis via CHOP activation [20,21].

The phosphoinositide 3-kinase (PI3K)/protein kinase B (AKT) signaling pathway is another key regulator of cell survival and function in AS. AKT signaling influences macrophage polarization and gene expression [22]. Previous studies have shown that ELA can activate the PI3K/AKT pathway, providing protection against oxidative stress and apoptosis in various cell types [23].

Despite the therapeutic potential of ELA-11, its short half-life, high immunogenicity, poor stability, and low bioavailability hinder its clinical application. Nanoparticle (NP)-based drug delivery systems offer a promising solution to these limitations [24]. In particular, pH-responsive NPs may exploit the acidic microenvironment of atherosclerotic plaques [25], facilitating targeted drug delivery.

This study aims to investigate the effects of ELA-11 on macrophage function and AS progression while elucidating the underlying molecular mechanisms. We hypothesized that ELA-11 attenuates AS by modulating macrophage function via the AKT-mediated ER stress pathway. To enhance its therapeutic efficacy, a pH-responsive NP delivery system, mPEG@ELA-11, was developed for targeted delivery to atherosclerotic plaques. Our findings provide valuable insights into the potential of ELA-11 as a novel therapeutic agent for AS and underscore the promise of NP-based drug delivery systems in cardiovascular diseases.

## Results

### Expression of ELA in human atherosclerotic plaques and serum

**Fig. 1.**
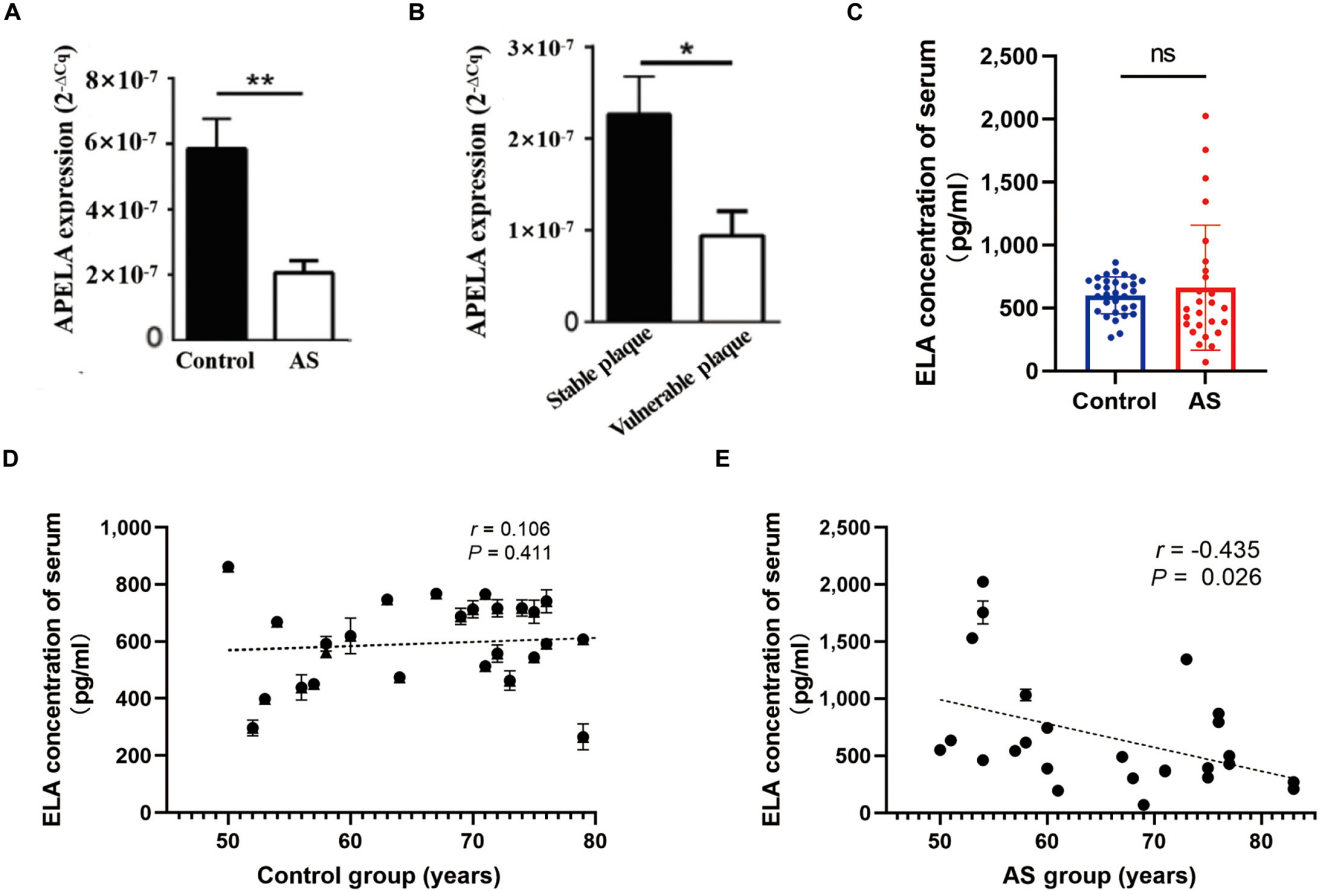
Expression of ELA in human atherosclerotic plaques and serum. (A) APELA mRNA expression in control arterial tissues versus atherosclerotic plaques detected by RT-qPCR (*n* = 6 per group). (B) APELA mRNA expression in stable versus unstable atherosclerotic plaques detected by RT-qPCR (*n* = 6 per group). (C) Comparison of serum ELA levels between patients with AS (*n* = 26) and healthy controls (*n* = 31, *P* = 0.58). (D and E) Simple linear regression analysis of serum ELA levels and age in healthy controls (D) and AS patients (E). Unpaired Student’s *t* test was used for pairwise comparisons in (A), (B), and (C). *P < 0.05, **P < 0.01; ns, not significant. AS, atherosclerosis.

To elucidate the role of ELA in AS, APELA gene expression, encoding ELA, was initially assessed in human carotid atherosclerotic plaques. Real-time quantitative PCR (RT-qPCR) analysis revealed a statistically significant down-regulation of APELA expression in plaque tissue compared with control arterial tissue (Fig. 1A). Notably, unstable plaques exhibited even lower APELA expression levels compared to stable plaques (Fig. 1B), suggesting a potential link between ELA expression and plaque stability.

Preoperative serum ELA levels in patients undergoing carotid endarterectomy (CEA) (this cohort was designated as the AS group) and healthy controls were subsequently evaluated using enzyme-linked immunosorbent assay (ELISA). In the AS group, a negative correlation between serum ELA levels and age was observed (Fig. 1E), a trend absent in the control group (Fig. 1D), indicating possible age-related dysregulation of ELA in patients with advanced AS. However, we did not observe a statistically significant difference in serum ELA levels between the AS group and the control group (Fig. 1C).

### In vitro effects of ELA-11 on macrophage function

Given the pivotal role of macrophages in AS progression, the effects of ELA-11 on key macrophage functions were evaluated in vitro. A foam cell model was first established by treating RAW264.7 macrophages with ox-LDL. Foam cells, characterized by their increased size and lipid droplet accumulation, were clearly visible under microscopic examination following ox-LDL exposure.

**Fig. 2.**
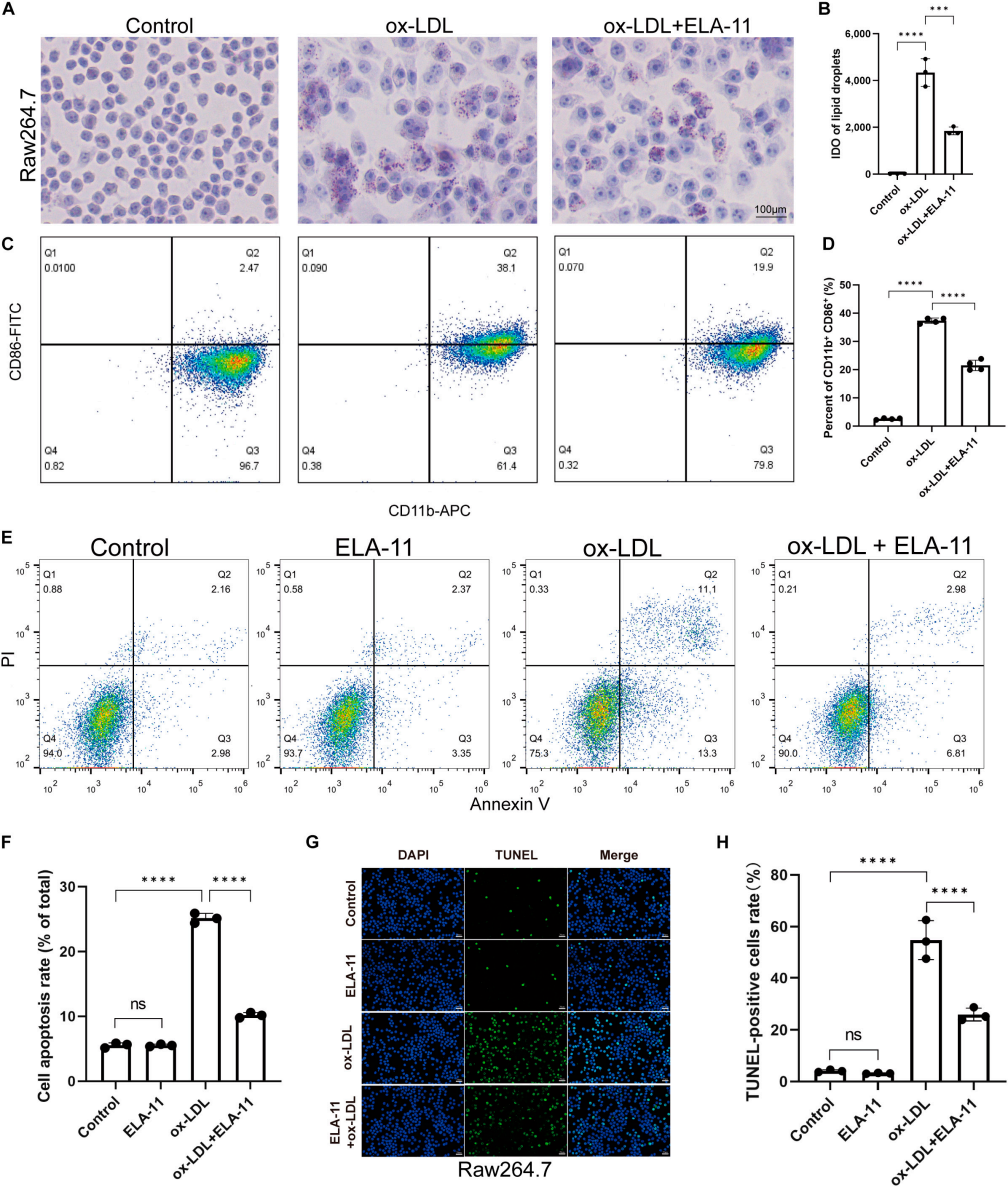
Effects of ELA-11 on macrophage function in vitro. (A) Representative Oil Red O staining showing lipid accumulation in RAW264.7 macrophages, scale bar = 100 μm. (B) Quantification of Oil Red O staining intensity (*n* = 3). (C) Flow cytometry analysis of M1 polarization markers (CD86 and CD11b) after ox-LDL (50 μg/ml, 48 h) exposure with/without ELA-11 (1 μM). (D) The histogram shows the percentage of Cd11b^+^ Cd86^+^ cells (*n* = 4). (E) Apoptosis assessed by Annexin V/PI staining and flow cytometry in macrophages. (F) The histogram shows the apoptosis rate of macrophages under different treatment conditions (*n* = 3). (G and H) TUNEL assay confirming reduced apoptosis with ELA-11 treatment (*n* = 3). Statistical analysis was conducted using one-way ANOVA with Tukey’s test for post hoc analysis. ***P < 0.001, and ****P < 0.0001.

To assess the impact of ELA-11 on foam cell formation, RAW264.7 macrophages were pretreated with ELA-11 (1 μM) for 2 h before being exposed to ox-LDL (50 μg/ml) for 48 h. Oil Red O staining revealed a significant reduction in the degree of lipid accumulation in macrophages treated with ELA-11 compared with those exposed to ox-LDL alone (Fig. 2A and B).

Next, the effect of ELA-11 on macrophage polarization was investigated, with a focus on the pro-inflammatory M1 phenotype. Flow cytometry analysis of CD86 and CD11b, markers of M1 polarization, showed that ELA-11 (1 μM) significantly inhibited ox-LDL-induced M1 polarization in RAW264.7 macrophages (Fig. 2C and D).

Finally, the anti-apoptotic effects of ELA-11 on ox-LDL-treated macrophages were examined. Annexin V/PI double staining and flow cytometry revealed that ELA-11 (1 μM) significantly reduced the percentage of apoptotic cells induced by ox-LDL (100 μg/ml) treatment (Fig. 2E and F). This protective effect was further corroborated by the TUNEL assay, which showed a marked reduction in TUNEL-positive cells in the ELA-11-treated group compared to the ox-LDL-only group (Fig. 2G and H).

Importantly, cell viability assays demonstrated that ELA-11, along with other compounds used in subsequent experiments (Tunicamycin and ML221), exhibited no significant cytotoxicity at the concentrations employed in these studies (Fig. S1).

These results collectively demonstrate that ELA-11 effectively mitigates multiple aspects of macrophage dysfunction linked to AS, including foam cell formation, M1 polarization, and apoptosis, while maintaining cell viability.

### Mechanism of ELA-11 action on macrophages

**Fig. 3.**
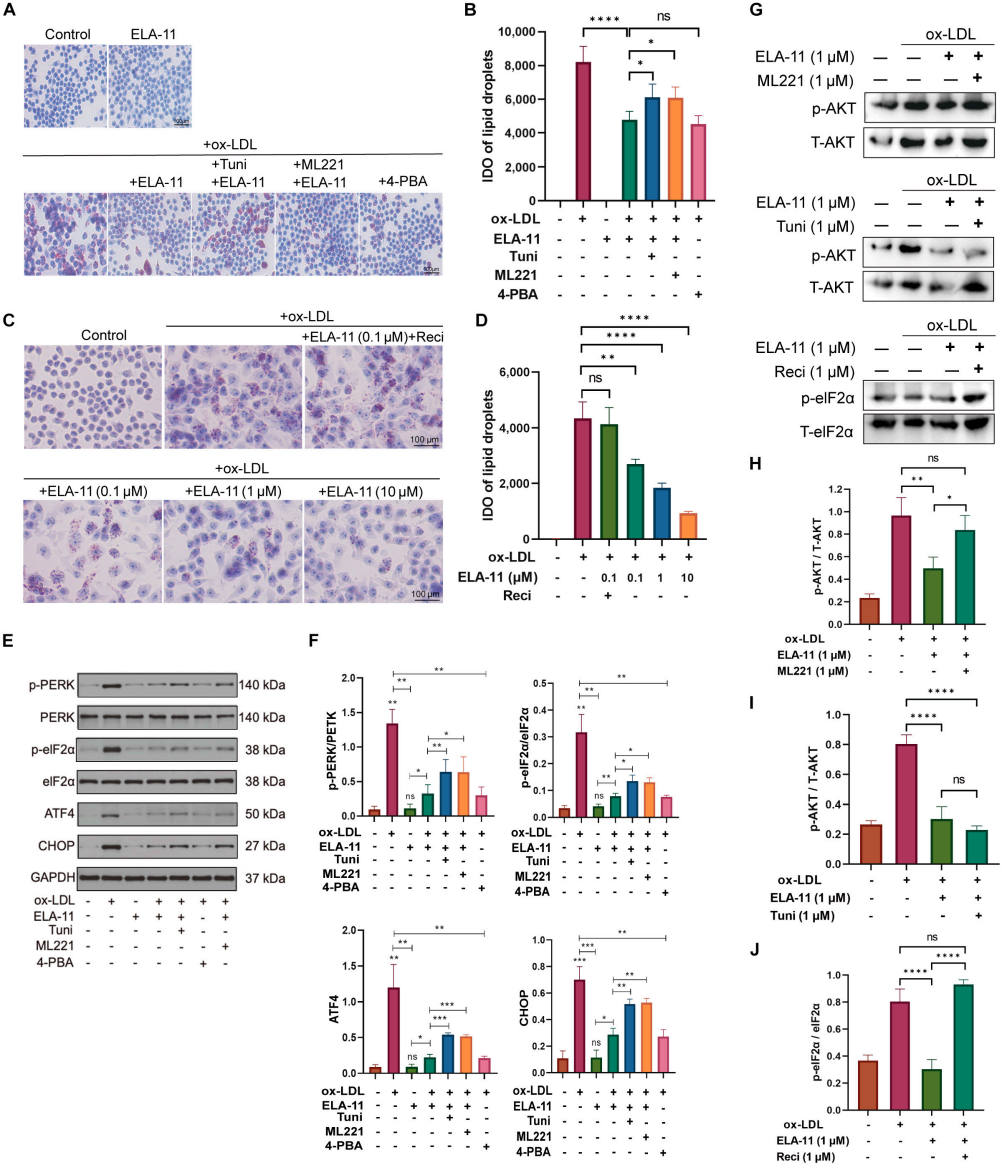
Mechanism of ELA-11’s effects on macrophages. (A) Representative Oil Red O staining showing lipid accumulation in RAW264.7 macrophages under different treatment conditions. (B) Quantification analysis of Oil Red O intensity. (C) Representative images and (D) quantification showing Recilisib (AKT activator, 1 μM) reverses ELA-11-mediated inhibition of foam cell formation and dose-dependent reduction in lipid accumulation by ELA-11. (E) Western blots and (F) quantification of ER stress markers: ox-LDL increases p-PERK, p-eIF2α, CHOP, and ATF4. ELA-11 suppresses these markers, effects reversed by Tunicamycin or ML221. (G) Western blot and (H to J) the histogram shows the relationship between AKT signaling and ER stress in the mechanism of ELA-11’s effects. Statistical analysis was conducted using one-way ANOVA with Tukey’s test for post hoc analysis. Each group, *n* = 3. *P < 0.05, **P < 0.01, ***P < 0.001, and ****P < 0.0001. Reci, Recilisib; Tuni, Tunicamycin; ML221, Apelin receptor antagonist; 4-PBA, 4-phenylbutyric acid.

Previous studies have suggested a link between ox-LDL-induced macrophage foam cell formation and apoptosis, and ER stress activation [26,27]. To investigate whether ELA-11 inhibits foam cell formation by alleviating ox-LDL-induced ER stress, rescue experiments were conducted in the foam cell model (Fig. 3A and B). Oil Red O staining demonstrated that ox-LDL (50 μg/ml) treatment for 48 h caused substantial cell swelling and lipid accumulation (*P* < 0.0001 vs. control). Cotreatment with ELA-11 significantly reduced foam cell formation (*P* < 0.0001 vs. ox-LDL group), with efficacy comparable to the ER stress inhibitor 4-PBA. Notably, the protective effect of ELA-11 was significantly attenuated by both the ER stress activator Tunicamycin (1 μM) and the APJ receptor antagonist ML221 (1 μM) (*P* < 0.05 vs. ox-LDL + ELA-11 group). Meanwhile, quantification of Oil Red O staining confirmed that ELA-11 decreased lipid accumulation in a dose-dependent manner (Fig. 3C and D).

Western blot analysis showed that while ox-LDL treatment significantly increased the expression of ER stress-associated proteins (p-PERK, p-eIF2α, CHOP, and ATF4), cotreatment with ELA-11 significantly reduced their relative levels (Fig. 3E and F). This suppression of ER stress by ELA-11 was diminished by ER stress activator Tunicamycin, indicating that the protective effects of ELA-11 are mediated through the ER stress pathway. Furthermore, cotreatment with ML221 might partially reverse ELA-11’s effects on ER stress markers, which could suggest that ELA-11 may modulate ER stress via the APJ receptor.

To explore whether the AKT pathway is involved in ELA-11’s effects, foam cells were treated with ELA-11 and the AKT activator Recilisib. Oil Red O staining revealed that Recilisib reversed ELA-11’s inhibitory effect on foam cell formation (Fig. 3C and D). Western blot analysis demonstrated that ELA-11 significantly reduced ox-LDL-induced AKT phosphorylation, and this effect was attenuated by ML221 (Fig. 3G and H), indicating that ELA-11 might modulate AKT signaling via the APJ receptor.

To further understand the relationship between AKT signaling and ER stress in the mechanism of ELA-11, additional experiments were conducted to test 3 potential scenarios: (a) ELA-11 independently inhibits both pathways via the APJ receptor, (b) ELA-11 affects ER stress through AKT inhibition, or (c) ELA-11 modulates AKT through ER stress inhibition. Cotreatment with ELA-11 and the ER stress activator Tunicamycin showed that ELA-11 maintained its inhibitory effect on AKT signaling (Fig. 3G and I), suggesting that ELA-11’s effect on AKT could be independent of ER stress. However, when AKT signaling was activated by Recilisib, ELA-11 lost its inhibitory effect on ER stress markers (Fig. 3G and J), indicating that ELA-11 could regulate ER stress through the AKT pathway.

Collectively, these results demonstrate that ELA-11 binds to the APJ receptor and subsequently inhibits AKT signaling, which leads to the suppression of ER stress, ultimately reducing macrophage foam cell formation. This hierarchical relationship between AKT signaling and ER stress provides novel insights into the molecular mechanisms underlying ELA-11’s therapeutic effects.

### In vivo effects of ELA-11 on AS in ApoE^-/-^ mice

**Fig. 4.**
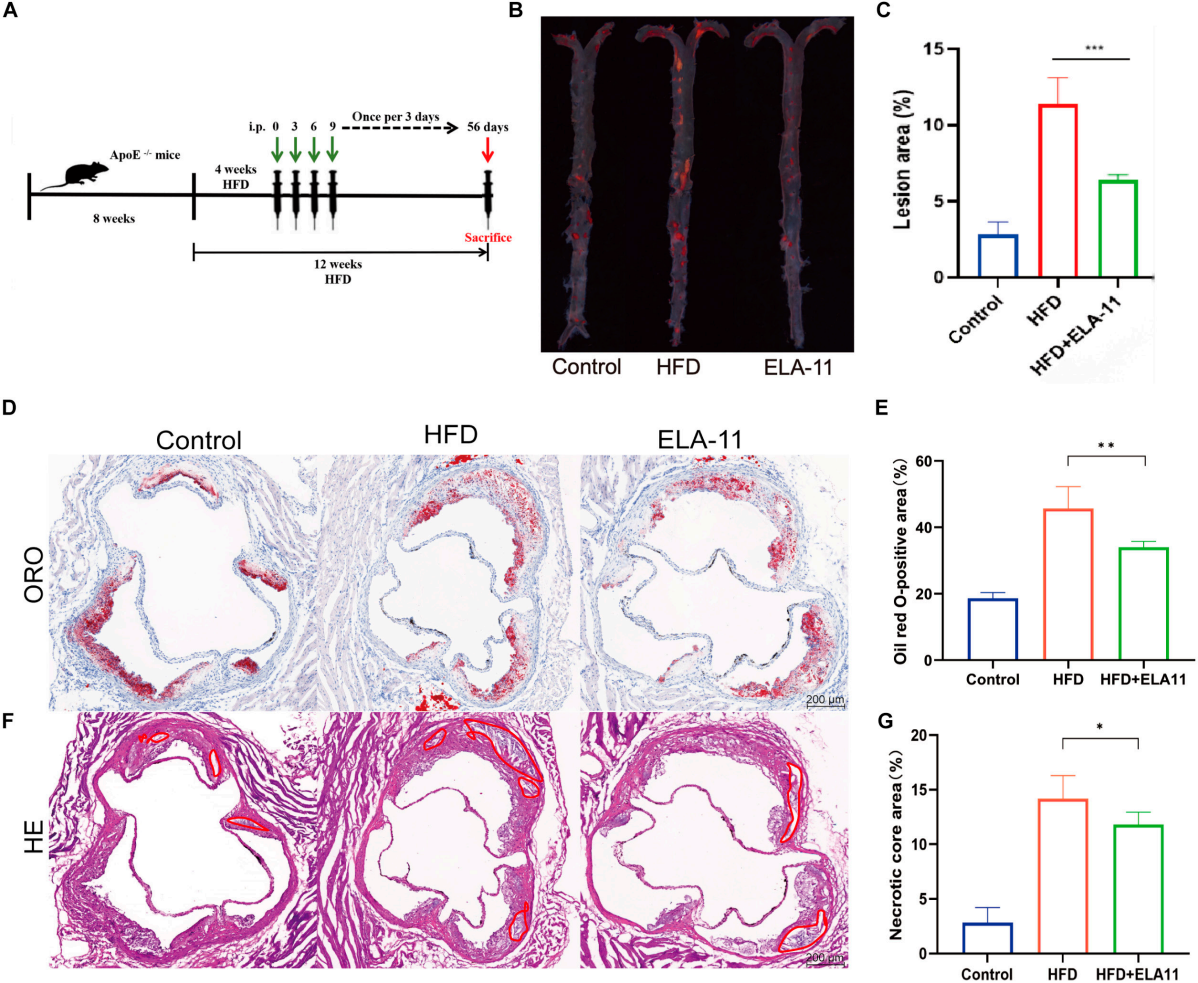
In vivo effects of ELA-11 in ApoE^-/-^ mice. (A) Experimental timeline. (B) Representative en face Oil Red O staining images of whole aortas. (C) Quantification of lesion area as percentage of total aortic surface (*n* = 6). (D) Oil Red O staining of aortic root cross-sections and (E) quantification (*n* = 3). (F) H&E staining of aortic root sections and (G) quantification of necrotic core area (*n* = 3). Statistical analysis was conducted using one-way ANOVA with Tukey’s test for post hoc analysis. *P < 0.05, **P < 0.01, and ***P < 0.001.

To evaluate the therapeutic efficacy of ELA-11 in AS, ApoE^-/-^ mice fed a high-fat diet (HFD) were utilized as an established AS model. Male ApoE^-/-^ mice were randomly allocated into 3 groups (*n* = 6 per group): a control group fed a normal chow diet (NCD), an HFD group treated with phosphate-buffered saline (HFD + PBS), and a treatment group treated with ELA-11 (HFD + ELA-11). ApoE^-/-^ mice were initiated on HFD at 8 weeks of age. Four weeks later, at the beginning of the fifth week of HFD feeding, mice in the HFD + ELA-11 group received intraperitoneal injections of ELA-11 (1 mg/kg) every 3 days for a total of 19 injections. Mice were sacrificed at 20 weeks of age (Fig. 4A).

After the treatment period, AS was assessed by examining the aorta. Oil Red O staining of the entire aorta revealed that the HFD + PBS group displayed the largest plaque area, covering approximately 11.42% of the aortic intimal surface. In contrast, ELA-11 treatment significantly reduced plaque area to 5.78% (Fig. 4B and C, *P* < 0.001), indicating a substantial attenuation of atherosclerotic progression.

Further analysis of plaque composition and stability was conducted through cross-sectional examination of the aortic root. Oil Red O staining of these sections confirmed a reduction in lipid deposition in the ELA-11-treated group relative to the HFD + PBS group (Fig. 4D, *P* < 0.01). Hematoxylin and eosin (H&E) staining revealed that the necrotic core area was significantly reduced in the ELA-11 group compared with the HFD group (Fig. 4E, *P* < 0.05). Collectively, these in vivo results demonstrate that ELA-11 treatment effectively mitigates atherosclerotic plaque formation, reduces necrotic core size, and preserves vascular wall integrity in ApoE^-/-^ mice. These results offer compelling evidence supporting the therapeutic potential of ELA-11 for treating AS.

### Development and characterization of mPEG@ELA-11 NPs

**Fig. 5.**
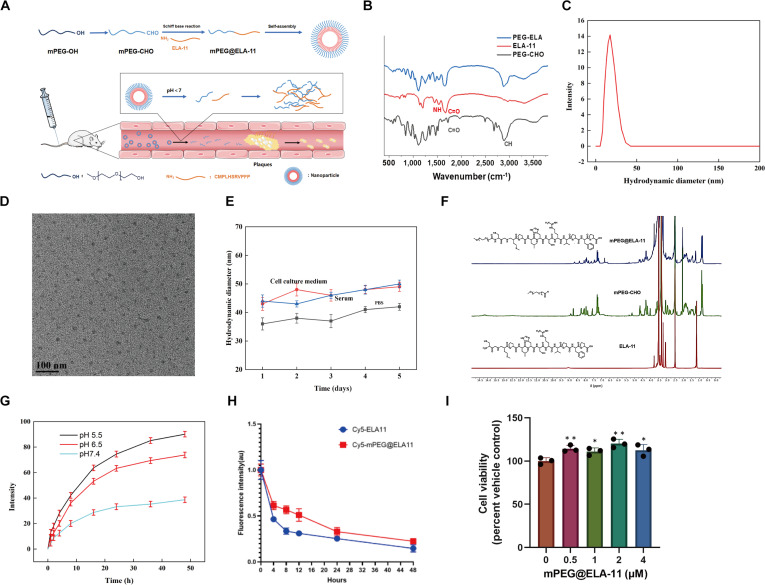
Characterization of mPEG@ELA-11 NPs. (A) Schematic illustration of synthesis and mechanism. (B) FT-IR spectra analysis. (C) DLS analysis showing hydrodynamic diameter of approximately 35 nm. (D) TEM image showing morphology (scale bar = 100 nm). (E) Colloidal stability study over 5 days in different media. (F) NMR spectra analysis. (G) pH-dependent release profile at pH 5.5, 6.5, and 7.4. (H) In vivo metabolism comparison between Cy5-ELA-11 and Cy5-mPEG@ELA-11. (I) Cell viability assay showing no significant cytotoxicity at different concentrations (*n* = 3). *P < 0.05, **P < 0.01.

To enhance the therapeutic efficacy of ELA-11 and achieve targeted delivery to atherosclerotic plaques, a pH-responsive NP system, mPEG@ELA-11, was developed. This system was engineered to prolong ELA-11’s circulation half-life while enabling its targeted release in the acidic microenvironment of atherosclerotic plaques. The synthesis of mPEG@ELA-11 involved conjugating methoxy polyethylene glycol (mPEG)—a hydrophilic polymer—to ELA-11 via a pH-sensitive Schiff base linkage (Fig. 5A). The successful formation of mPEG@ELA-11 was confirmed through a range of characterization techniques.

Fourier-transform infrared (FT-IR) spectroscopy analysis demonstrated the presence of characteristic peaks from both mPEG and ELA-11 in the mPEG@ELA-11 spectrum (Fig. 5B). Notably, the peak at 1,680 cm^−1^, corresponding to the carbonyl (C=O) stretching vibration of mPEG-CHO, and the peak at 1,630 cm^−1^, indicative of the amide bond of ELA-11, were both observed, confirming successful conjugation. Dynamic light scattering (DLS) analysis revealed that mPEG@ELA-11 NPs exhibited a hydrodynamic diameter of ~35 nm in PBS (Fig. 5C), indicating excellent solubility and suitability for biological applications. Transmission electron microscopy (TEM) revealed a well-defined, monodispersed layered structure with an approximate size of ~25 nm (Fig. 5D). The slight difference between DLS and TEM measurements is expected, as DLS measures the hydrodynamic diameter in solution, while TEM visualizes dehydrated NPs.

The colloidal stability of mPEG@ELA-11 was evaluated in various physiological media over a 5-day period. The hydrodynamic diameter remained stable in PBS, serum, and 1640 culture medium, with only minor increases observed over time (Fig. 5E). On day 1, the sizes in PBS, serum, and 1640 medium were 36 ± 2.12, 43 ± 3.01, and 44 ± 4.53 nm, respectively. By day 5, these had increased slightly to 42 ± 3.36, 49 ± 3.49, and 50 ± 2.98 nm, respectively. These results suggest that mPEG@ELA-11 NPs maintain their structural integrity under physiological conditions, a key property for in vivo applications.

Nuclear magnetic resonance (NMR) spectroscopy further validated the synthesis of mPEG@ELA-11. The disappearance of the characteristic aldehyde peak of mPEG-CHO in the mPEG@ELA-11 spectrum, alongside the presence of peaks corresponding to both mPEG-CHO and ELA-11, substantiated the complete reaction and successful preparation of mPEG@ELA-11 (Fig. 5F).

To assess the pH-responsive release behavior of mPEG@ELA-11, in vitro release studies were conducted. The results demonstrated that mPEG@ELA-11 exhibited a pH-dependent release of ELA-11, with 91.2% release at pH 5.5, 74.1% at pH 6.5, and only 38.7% at pH 7.4 after 48 h (Fig. 5G). This pH-sensitive release profile is advantageous for targeting atherosclerotic plaques, which typically exhibit a lower pH than normal arterial tissue. As shown in Fig. 5H, Cy5-mPEG@ELA-11 demonstrated significantly slower metabolism compared to Cy5-ELA-11 in mice. At 12 h post-injection, only 45% of Cy5-mPEG@ELA-11 was degraded, while 70% of Cy5-ELA-11 had already been degraded. At 48 h, the degradation rate of Cy5-mPEG@ELA-11 remained lower than that of Cy5-ELA-11. This indicates that mPEG@ELA-11 effectively increased the in vivo circulation half-life of ELA-11.

Finally, the cytotoxicity of mPEG@ELA-11 was assessed using the CCK-8 assay. RAW264.7 macrophages treated with various concentrations of mPEG@ELA-11 (0.5, 1, 2, and 4 μM) exhibited no significant reduction in cell viability compared to untreated controls (Fig. 5I). The absence of cytotoxicity is a critical factor for the potential clinical application of mPEG@ELA-11.

In summary, the mPEG@ELA-11 NPs system was successfully developed and characterized, exhibiting desirable physicochemical properties such as appropriate size, pH-responsive release, good colloidal stability, and low cytotoxicity. These features position mPEG@ELA-11 as a promising candidate for the targeted delivery of ELA-11 to atherosclerotic plaques.

### Biodistribution of mPEG@ELA-11 NPs

**Fig. 6.**
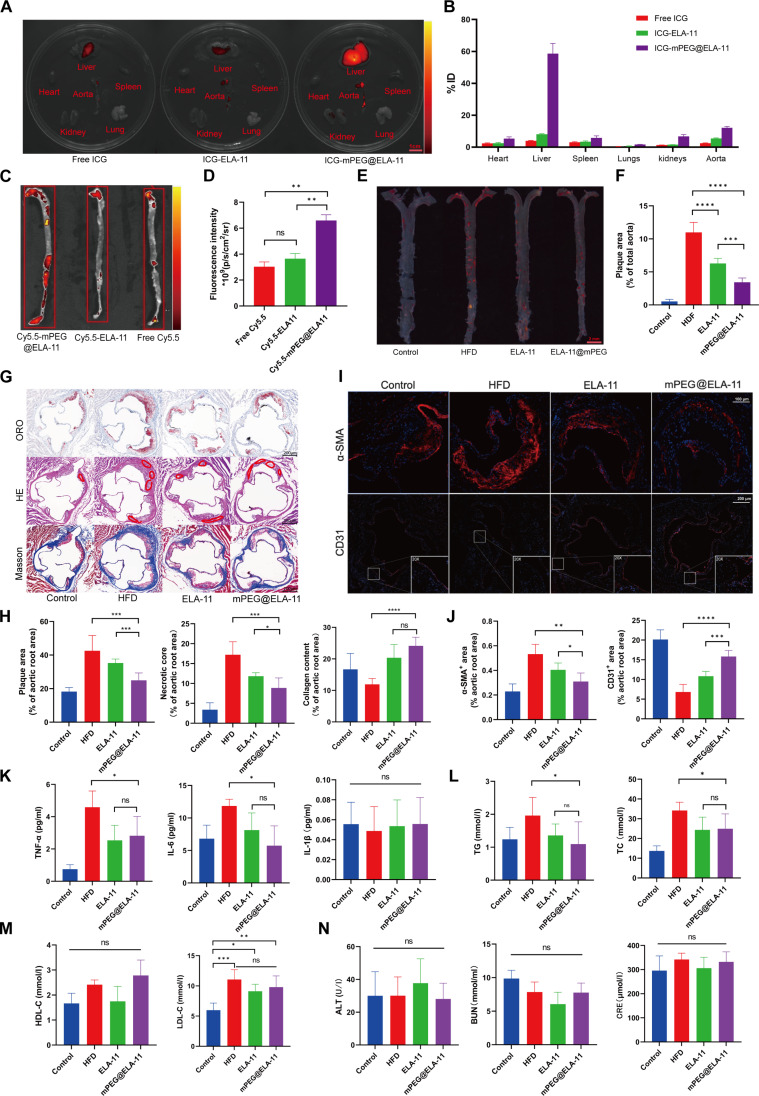
In vivo efficacy of mPEG@ELA-11. (A) Ex vivo fluorescence imaging and (B) quantitative analysis of ICG fluorescent signal in mice organs. Biodistribution of mPEG@ELA-11 NPs at 24 h post-injection, expresses as percentage of injected dose (*n* = 6). (C) Ex vivo fluorescence imaging and (D) quantitative analysis of Cy5.5 fluorescent signal in aortas. ApoE^-/-^ mice fed with high-fat food for 3 months were i.v. administered with Cy5.5, Cy5.5-ELA-11, and Cy5.5-mPEG@ELA-11 NPs, respectively. Aorta tissues from different mice (*n* = 3). (E) En face Oil Red O staining and (F) quantitative analysis of aortas from all treatment groups (*n* = 6). (G) Histological analysis of aortic roots including Oil Red O, H&E, and Masson’s trichrome staining. (H) Analysis of plaque area in total aortas, plaque area, necrotic core areas, and collagen content in aortic root (*n* = 6). (I) Immunofluorescence staining showing α-SMA and CD31 expression. (J) Quantification of α-SMA and CD31 expression (*n* = 6). (K) The levels of TNF-α, IL-6, and IL-1β in blood serum (*n* = 6). (L to N) Comprehensive blood biochemistry analysis (*n* = 6). All data presented as mean ± SD. Statistical analysis was conducted using one-way ANOVA with Tukey’s test for post hoc analysis. *P < 0.05, **P < 0.01, ***P < 0.001, and ****P < 0.0001.

ICG (indocyanine green)-labeled mPEG@ELA-11 NPs were employed to evaluate the NPs biodistribution ex vivo. The fluorescence images of the major organs (including aorta) were harvested from the mice at 24 h. As shown in Fig. 6A and B, substantial accumulation was observed in the liver (58.7 ± 6.3% ID) and aorta (12.1 ± 0.8% ID), and low nontarget uptake was detected in other critical organs (spleen, kidneys, heart, and lungs). By contrast, when compared with the ICG-ELA-11 and free ICG treatments, mice administered ICG-mPEG@ELA-11 NPs showed the strongest fluorescence signal in liver and aorta. These data demonstrate that mPEG@ELA-11 can preferentially be delivered to atherosclerotic plaque areas.

### In vivo efficacy of mPEG@ELA-11 NPs

To evaluate the therapeutic potential of mPEG@ELA-11 NPs in vivo, a series of experiments were conducted using ApoE^-/-^ mice fed an HFD for 3 months to induce AS. The mice were randomly assigned to 4 groups (*n* = 6): a negative control group fed an NCD, an HFD group treated with PBS (HFD + PBS), an HFD group treated with free ELA-11 (HFD + ELA-11), and an HFD group treated with mPEG@ELA-11 NPs (HFD + mPEG@ELA-11). The administration route, frequency, and dosage were the same as described above.

To assess the targeting efficiency of mPEG@ELA-11 to atherosclerotic plaques, Cy5.5-mPEG@ELA-11, Cy5.5-ELA-11, and free Cy5.5 were labeled with Cy5.5 fluorescent dye and intravenously administered to ApoE^-/-^ mice that had been on an HFD for 3 months. After 24 h, ex vivo fluorescence imaging was performed on the aorta. Notably, Cy5.5-labeled mPEG@ELA-11 exhibited significantly enhanced accumulation in the aorta compared to both free Cy5.5 and Cy5.5-ELA-11 (Fig. 6C and D, *P* < 0.01). This observation indicates the superior targeting ability of mPEG@ELA-11 to atherosclerotic lesions, likely due to the combined effects of the enhanced permeability and retention (EPR) phenomenon and the pH-sensitive properties of the NPs.

Next, the therapeutic efficacy of mPEG@ELA-11 was compared to that of free ELA-11. Oil Red O staining of the entire aorta revealed a greater reduction in atherosclerotic plaque area following mPEG@ELA-11 treatment compared to free ELA-11 (Fig. 6E and F, *P* < 0.001). This enhanced therapeutic effect was further validated by analysis of aortic root sections. Oil Red O and H&E staining showed that mPEG@ELA-11 treatment resulted in a more substantial decrease in both the overall plaque area and necrotic core size compared to free ELA-11 (Fig. 6G and H, *P* < 0.001, *P* < 0.05). Masson’s trichrome staining of aortic root sections revealed that both ELA-11 and mPEG@ELA-11 treatments increased collagen content within the plaques, indicating enhanced plaque stability (Fig. 6G and H, *P* < 0.0001). However, no significant difference was observed between the 2 treatment groups in terms of collagen content. Immunofluorescence staining for α-SMA demonstrated that mPEG@ELA-11 significantly inhibited smooth muscle cell proliferation in the plaque area when compared to the ELA-11 and HFD + PBS group (Fig. 6I and J, *P* < 0.05, *P* < 0.01). Notably, the inhibitory effect of the mPEG@ELA-11 group was more pronounced than that of the ELA-11 group (*P* < 0.05). CD31 immunofluorescence staining suggested that mPEG@ELA-11 treatment was more effective than free ELA-11 in preserving the integrity of the vascular endothelium. The endothelial layer appeared more continuous and intact in the mPEG@ELA-11 group compared to both the ELA-11 and HFD + PBS groups (Fig. 6I and J, *P* < 0.001, *P* < 0.0001).

### Biosafety evaluation of mPEG@ELA-11 NPs

To evaluate the safety and biocompatibility of the treatments, histological analysis was performed on major organs (heart, liver, spleen, lungs, and kidneys) from all groups. No significant histopathological changes in any of the organs or abnormal changes in body weight were observed in any of the organs from mice treated with ELA-11 or mPEG@ELA-11 (Figs. S2 and S3), indicating favorable biocompatibility for both treatments. Inflammatory markers tumor necrosis factor-α (TNF-α) and interleukin-6 (IL-6) were significantly lower in the ELA-11 and mPEG@ELA-11 groups compared to the HFD group (Fig. 6K, *P* < 0.05), while IL-1β levels showed no significant differences among the groups. Notably, the HFD group exhibited significantly higher levels of total cholesterol, triglycerides, and LDL-C compared to both ELA-11 and mPEG@ELA-11 treatment groups (Fig. 6L and M, *P* < 0.05). Blood biochemistry analysis showed no significant differences in liver function markers (alanine aminotransferase) or kidney function markers (blood urea nitrogen and creatinine) between the treatment groups and controls (Fig. 6N).

In summary, these in vivo results demonstrate that mPEG@ELA-11 NPs offer enhanced targeting to atherosclerotic plaques and superior therapeutic efficacy compared to free ELA-11. The NP formulation more effectively reduced plaque area, decreased necrotic core size, and preserved vascular endothelial integrity. Furthermore, both ELA-11 and mPEG@ELA-11 treatments exhibited good safety profiles and potential systemic benefits, including improved lipid profiles and reduced inflammation. These results highlight the promising potential of mPEG@ELA-11 as a novel nanomedicine approach for the treatment of AS.

## Discussion

Herein, we provide novel insights into the role of ELA-11 in AS and its therapeutic potential. We report, for the first time, that ELA-11 exerts protective effects against AS by modulating macrophage function via the AKT-mediated ER stress pathway. Furthermore, we developed a pH-responsive NP delivery system, mPEG@ELA-11, which significantly enhances the in vivo therapeutic efficacy of ELA-11.

Initial findings indicated a significant down-regulation of APELA gene expression, encoding ELA, in human carotid atherosclerotic plaques compared to control arterial tissue. Notably, this down-regulation was more pronounced in unstable plaques, suggesting an association between ELA expression and plaque stability. The observed age-related decline in serum ELA levels in the AS group hints at a potential role for ELA in the age-dependent progression of AS, underscoring the need for further investigation into its contribution to the pathogenesis of this disease. While clear differences in local APELA expression were observed, serum ELA levels did not directly correlate with the presence or severity of carotid AS. This inconsistency may be attributed to the dissociated regulatory patterns of systemic and local ELA. ELA peptide expression in the human cardiovascular system has been reported to be restricted to vascular endothelial cells [28]. Serum ELA is maintained by systemic synthesis, degradation, and clearance—even in AS, compensatory secretion from unaffected tissues preserves this balance. In contrast, intraplaque ELA depends on local dynamics: AS plaques exhibit reduced ELA synthesis (due to endothelial dysfunction) and increased consumption (via inflammatory proteases), lowering local levels. Previous studies have shown that ELA levels are reduced in patients with essential hypertension, pulmonary arterial hypertension, and chronic coronary occlusion, yet elevated in patients with acute ST-segment elevation myocardial infarction and acute coronary syndrome—with levels correlating with the severity of coronary stenosis [28–32]. These findings collectively indicate that ELA peptide expression in vivo is modulated by multiple comprehensive factors, further supporting the notion that serum ELA levels may not simply reflect local changes in atherosclerotic plaques. In our study, no significant difference in serum ELA concentration was found between patients with AS and healthy controls, and serum ELA peptide levels in the AS group were relatively scattered. This variability might be associated with clinical heterogeneity including disease severity, comorbidities (e.g., hypertension and diabetes), and preoperative medication differences. These results highlight the need for further investigation into the relationship between circulating ELA levels and local tissue expression in the context of AS. Therefore, future studies should expand sample size (to reduce outlier impact) and conduct stratified analyses (by age, stenosis, comorbidities, or medications) to clarify serum ELA dynamics.

The mechanism underlying ELA-11’s protective effects involves modulation of both the AKT signaling pathway and the ER stress response. Results revealed that ELA-11 suppressed ox-LDL-induced activation of the AKT pathway and down-regulated key ER stress markers, including p-PERK, p-eIF2α, CHOP, and ATF4. These findings align with previous studies implicating AKT signaling [22] and ER stress [17,33] in AS progression. The interplay between AKT signaling and ER stress in the context of ELA-11 treatment is particularly noteworthy. Our preliminary evidence indicating that ELA-11 modulates ER stress predominantly via the AKT pathway, as opposed to the inverse direction. This proposed hierarchical relationship could provide new perspectives on the molecular mechanisms driving ELA-11’s protective role in AS, though its definitive relevance remains to be verified. Chen et al. [34] found that in DOCA/salt-induced hypertension, renal ELA overexpression alleviates hypertension and renal injury by inhibiting the renal NADPH oxidase/ROS/NLRP3 inflammasome pathway, and this effect is not affected by APJ deficiency, indicating that ELA may act via an APJ-independent mechanism in this context. Another study utilizing CRISPR/Cas9 gene-editing technology in human embryonic stem cells (hESCs) found that even after knocking out APJ, exogenously added ELA N-terminal fluorescent protein could still enter the cells, indicating the existence of non-APJ receptors for ELA in hESCs. To confirm whether such non-APJ receptor-dependent pathways are involved in the anti-atherosclerotic effects of ELA and its degradation fragments in macrophages, additional experiments are needed.

The in vivo efficacy of ELA-11 in reducing atherosclerotic plaque burden and improving plaque stability in ApoE^-/-^ mice provides compelling evidence for its therapeutic potential. The observed reductions in plaque area, necrotic core size, and inflammatory markers, alongside increased collagen content, suggest that ELA-11 may not only retard the progression of AS but also promote the stabilization of existing plaques. This dual action is particularly advantageous from a clinical perspective, as it addresses both the prevention of new atherosclerotic lesions and the management of preexisting plaques. Despite these promising outcomes, the short half-life and poor bioavailability of ELA-11 pose substantial challenges to its clinical application. To overcome these limitations, a pH-responsive NP delivery system, mPEG@ELA-11, was developed. This strategy leverages the acidic microenvironment in atherosclerotic plaques [25] for targeted drug delivery.

Polyethylene glycol (PEG) finds extensive application in the surface modification of particles. This is attributed to its hydrophilic properties and biocompatibility, along with its ability to enhance the colloidal stability of particles and prolong their circulation time in the bloodstream. Studies have indicated that PEGylation can reduce the adsorption of proteins onto different types of particles [35]. Beyond their ability to repel proteins, PEG-modified particles also exhibit greater colloidal stability and a reduced rate of nonspecific cellular uptake. In our study, the mPEG@ELA-11 NPs exhibited several favorable properties, including optimal size (approximately 35 nm), a pH-responsive release profile, and excellent colloidal stability. Enhanced accumulation of mPEG@ELA-11 in atherosclerotic plaques, as demonstrated by in vivo imaging studies, can be attributed to both the EPR effect and the NPs’ pH-responsive characteristics. However, mPEG@ELA-11 exhibits considerable accumulation in the liver, which may hinder its therapeutic efficacy and raise concerns about potential off-target toxicity. To address this issue, refinement of the PEGylation strategy (including optimizing the molar ratio of mPEG to ELA-11, adopting branched mPEG, or using mPEG with a slightly higher molecular weight) could be effective approaches to reduce the off-target accumulation of mPEG@ELA-11 in the liver, thereby improving its targeting specificity and therapeutic safety [36].

The superior therapeutic efficacy of mPEG@ELA-11 relative to free ELA-11 in our in vivo studies underscores the potential of this NP-based approach. The more pronounced reductions in plaque area and necrotic core size, coupled with improved preservation of vascular endothelial integrity, highlight the benefits of targeted and sustained delivery of ELA-11 to atherosclerotic lesions. While both ELA-11 and mPEG@ELA-11 demonstrated favorable safety profiles in this study, the long-term safety and potential immunogenicity of NP-based therapies remain critical considerations for clinical translation. Future research should focus on evaluating the chronic effects and possible side effects of long-term mPEG@ELA-11 administration. Additionally, the observed systemic benefits of ELA-11 and mPEG@ELA-11 treatment, including improvements in lipid profiles and reductions in inflammatory markers, suggest that these therapies may offer broader cardiovascular benefits beyond their localized effects on atherosclerotic plaques. This is particularly noteworthy given the growing recognition of AS as a systemic inflammatory disease.

Additionally, the translational potential of mPEG@ELA-11 hinges on addressing its synthesis scalability and regulatory compliance, which require further exploration. Currently, mPEG@ELA-11 is synthesized via Schiff base reaction between mPEG-CHO and ELA-11 (1:1 molar ratio, dimethyl sulfoxide [DMSO], 24 h) followed by water self-assembly, dialysis purification (3 days), and freeze–drying, with ~67.4% yield. Scalability needs optimization: dialysis will be replaced by tangential flow filtration to cut time to <8 h and boost yield >80%; automated systems and in-process FT-IR/DLS monitoring will reduce batch variability (current CV ~8% to 10%). Regulatory hurdles include expanding characterization to 12-month stability data and protein corona analysis, and supplementing GLP-toxicity and anti-ELA/PEG antibody detection for safety. These steps are critical to advance mPEG@ELA-11’s clinical translation.

## Conclusion

Our study, for the first time, demonstrates the therapeutic potential of ELA-11 in AS. The development of mPEG@ELA-11 NPs presents a promising strategy for enhancing the therapeutic efficacy of ELA-11 through targeted delivery to atherosclerotic plaques.

## Materials and Methods

### Clinical sample collection and processing

#### Human samples

Atherosclerotic plaque specimens were collected from 26 patients who underwent CEA at the Department of Vascular Surgery, Shanghai Changzheng Hospital, between September 2021 and February 2023. For control specimens, 6 internal mammary artery specimens were obtained from patients undergoing coronary artery bypass graft surgery. Additionally, peripheral blood samples were collected from 2 groups: the aforementioned CEA patients prior to their CEA surgery, and 31 healthy volunteers at the time of their health examination at the hospital’s health examination center. This study was approved by the hospital’s Ethics Committee (Approval No. SC090966) and conducted in accordance with the principles of the Declaration of Helsinki. All participants provided written informed consent prior to their participation in the study.

### Specimen processing

Fresh plaque specimens were immediately placed in ice-cold PBS containing 25 mM EDTA and protease/phosphatase inhibitors. Unstable plaques (Stary type IV, V, or VI) were identified and sectioned longitudinally along the carotid artery axis. After washing with cold PBS, plaques were examined for signs of rupture, typically located near the carotid bifurcation. Ruptured plaque regions (1 cm × 1 cm) were collected along with adjacent tissue. For stable plaque regions (Stary types I and II), comparable-sized tissue samples were collected from the proximal and distal ends of the same specimen. One-millimeter-thick sections from each specimen were fixed in 10% formalin for histological analysis, while the remaining tissue was snap-frozen for subsequent molecular studies.

### Cell culture and treatment

#### Cell lines

RAW264.7 murine macrophages (Cobioer Biosciences Co., Ltd., China) were maintained in high-glucose Dulbecco’s Modified Eagle Medium supplemented with 10% fetal bovine serum and 1% penicillin/streptomycin at 37 °C in a humidified atmosphere with 5% CO_2_. THP-1 monocytes (Cobioer Biosciences Co., Ltd., China) were cultured in RPMI-1640 medium and differentiated into macrophages using 100 ng/ml phorbol-12-myristate-13-acetate (PMA, MedChemExpress HY-18739) for 48 h.

### Foam cell formation and drug treatment

For foam cell formation assays, cells were seeded in 6-well plates at 1 × 10^6^ cells/well. Cells were pretreated with ELA-11 (1 μM) for 2 h before exposure to oxidized low-density lipoprotein (ox-LDL, 50 or 100 μg/ml) for 48 h. Where indicated, cells were cotreated with tunicamycin (1 μM, ER stress activator), ML221 (1 μM, APJ receptor antagonist), Recilisib (1 μM, AKT activator), or 4-PBA (1 μM, ER stress inhibitor).

### Cellular assays

#### Oil Red O staining

Cells were fixed with 4% paraformaldehyde for 30 min, rinsed with 60% isopropanol, and stained with Oil Red O working solution for 10 min. After washing with distilled water, stained cells were imaged using a light microscope. For quantification, staining intensity was analyzed using ImageJ software.

#### Cell viability assay

Cell viability was assessed using the CCK-8 kit (Biosharp, China) according to the manufacturer’s instructions. Cells were seeded in 96-well plates at 1 × 10^5^ cells/ml and treated with various compounds for 24 to 72 h before analysis.

#### Apoptosis analysis

Apoptosis was evaluated using both TUNEL assay and Annexin V/PI staining. For the TUNEL assay (ServiceBio, China), cells were fixed, permeabilized, and incubated with TdT enzyme and CF488-dUTP label overnight at 4 °C. For Annexin V/PI staining (Tianjin Sungene Biotech), cells were stained with FITC-Annexin V and PI for 15 min and analyzed by flow cytometry (CytoFlex, Beckman).

#### Flow cytometry analysis

For macrophage polarization analysis, cells were stained with fluorochrome-conjugated antibodies targeting CD11b and CD86. Data acquisition was performed using a CytoFlex flow cytometer, and analysis was conducted using FlowJo software.

### Molecular and biochemical analyses

#### Western blot analysis

Cells were lysed in RIPA buffer supplemented with protease and phosphatase inhibitors. Equal protein amounts were separated by 10% sodium dodecyl sulfate–polyacrylamide gel electrophoresis, followed by transfer to polyvinylidene fluoride membranes. Membranes were blocked with 5% milk and incubated overnight at 4 °C with primary antibodies against p-PERK, PERK, p-eIF2α, eIF2α, ATF4, CHOP, p-AKT, AKT, and GAPDH, followed by horseradish peroxidase-conjugated secondary antibodies. Protein bands were visualized using enhanced chemiluminescence reagents.

#### RT-qPCR analysis

Total RNA was extracted from tissue samples using TRIzol reagent and the PureLink RNA Mini Kit. RNA (1 μg) was reverse-transcribed using the Promega reverse transcription system. Real-time PCR was performed with TaqMan primers for human APELA and 18S rRNA on an ABI 7500 system.

### ELISA

Serum ELA levels were quantified using commercial ELISA kits according to the manufacturer’s instructions, with samples diluted 1:2 in assay buffer and absorbance measured at 450 nm.

### Synthesis and characterization of mPEG@ELA-11

#### Synthesis

The synthesis of mPEG@ELA-11 involved conjugating 100 mg of ELA-11 with 78 mg of mPEG-CHO in DMSO at room temperature for 24 h. The resulting product was self-assembled in ultrapure water, purified by dialysis (molecular weight cutoff: 2,000 Da), and lyophilized to yield a white powder.

#### Characterization

NP characterization was performed using FT-IR, NMR, DLS, and TEM. pH-responsive drug release was evaluated at pH values of 5.5, 6.5, and 7.4. For fluorescence imaging, NPs were labeled with ICG, Cy5 or Cy5.5.

### Animal studies

#### Animals and treatment

Male ApoE^-/-^ mice (26 to 31 g, 8 weeks old) were obtained from GemPharmatech Co., Ltd. and housed under specific pathogen-free conditions. Mice were randomly allocated into 4 groups (*n* = 6/group): NCD, HFD + PBS, HFD + ELA-11, and HFD + mPEG@ELA-11. The HFD (Research Diets D12079B) consisted of 21% fat and 0.21% cholesterol. Drug treatments (1 mg/kg) were administered intraperitoneally every 3 days for 8 weeks, beginning at week 13. All animal procedures were approved by the institutional animal care committee.

#### Tissue collection and analysis

At the study endpoint, mice were euthanized under isoflurane anesthesia. Blood was collected for biochemical analysis, and the entire aorta was harvested from the ascending aorta to the iliac bifurcation. For en face analysis, aortas were stained with Oil Red O. The aortic root was sectioned for histological analysis, including Oil Red O, H&E, Masson’s trichrome, and immunofluorescence staining for CD31 and α-SMA.

#### Imaging studies

For biodistribution studies, ICG-labeled formulations were administered intravenously, and ex vivo fluorescence imaging of organs was performed using Small Animal Imaging System (Tanon ABL-X6) after 24 h. To specifically investigate the targeting efficacy of mPEG@ELA-11, Cy5.5 fluorescent labeling was employed. Tissue sections for histological analysis were imaged using a fluorescence microscope (Leica DM2500).

### Statistical analysis

Data are presented as mean ± standard deviation. Statistical analyses were conducted using GraphPad Prism 9.0. Two-group comparisons were performed using Student’s *t* test, while multiple group comparisons were analyzed by one-way ANOVA with Tukey’s test. A *P* value < 0.05 was considered statistically significant. For analyzing the correlation of serum ELA levels, simple linear regression analysis was performed.

## Data Availability

The data that support the findings of this study are available from the corresponding authors upon reasonable request.
