# Supplementary material for: mPEG@ELA-11 Alleviates Atherosclerosis via AKT-ER Stress-Mediated Macrophage Modulation
Source: BME Front. 2025 Nov 25;6:0203. doi: 10.34133/bmef.0203 (PMC12645589; doi:10.34133/bmef.0203)
Supplement: Supplementary 1 — Figs. S1 to S3 [file bmef.0203.f1.zip › Supplementary Materials.docx]

**Supplementary Material**

**mPEG@ELA-11 Alleviates Atherosclerosis via AKT-ER Stress-Mediated Macrophage Modulation**

Xiaoguang Li, Ning Dou, Linshan Zhong, Yicheng Wu, ZhenZhen Cai, Zaixu Zhao, Lefeng Qu, Qixia Jiang

**Fig. S1. Effect of ELA11, Tuni and ML221 on the viability of ox-LDL-induced macrophages (Raw264.7)**


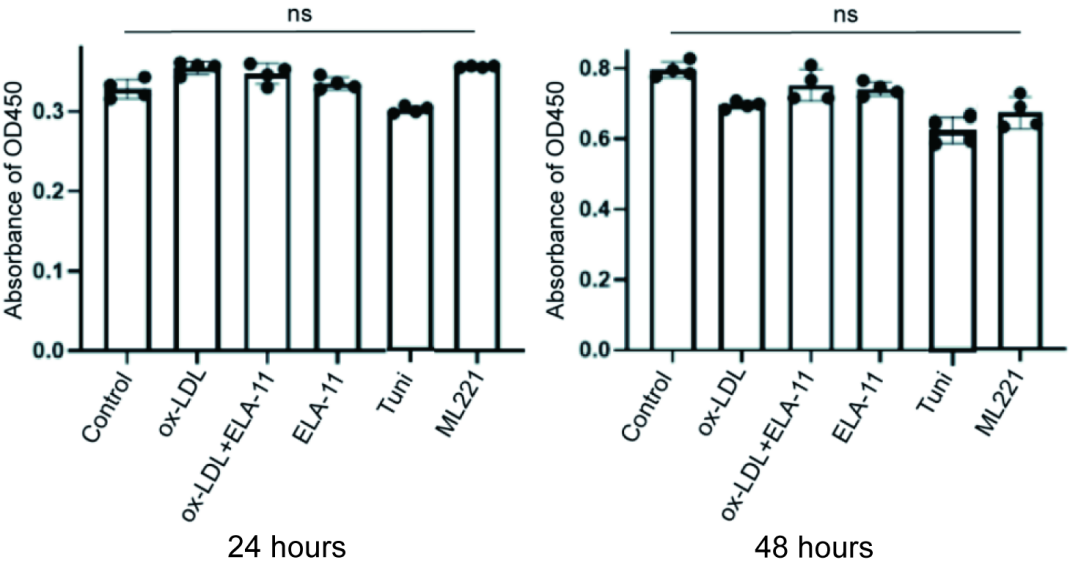


CCK-8 cell viability assay results at 24 and 48 hours showing no significant cytotoxicity among groups (n=4, p > 0.05).

**Fig. S2. Changes of mice body weight.**

Body weight of mice from various treatment groups throughout the treatment period. No statistical significance was observed between groups (n=6, p＞0.05).


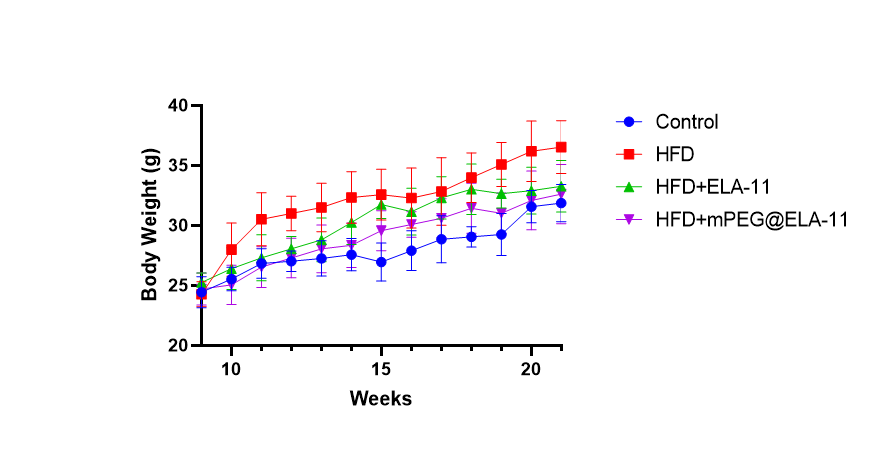

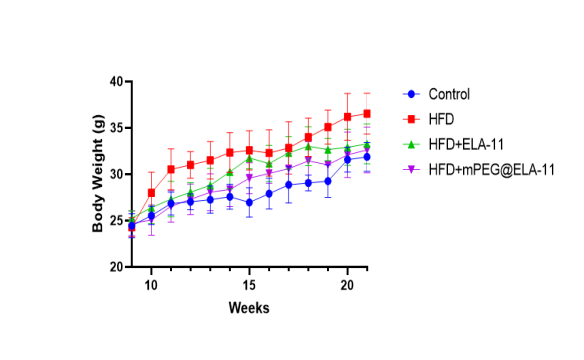


**Fig. S3. Histological evaluation of ELA-11 and mPEG@ELA-11 long-term** **toxicity in ApoE-/- mice.**

**
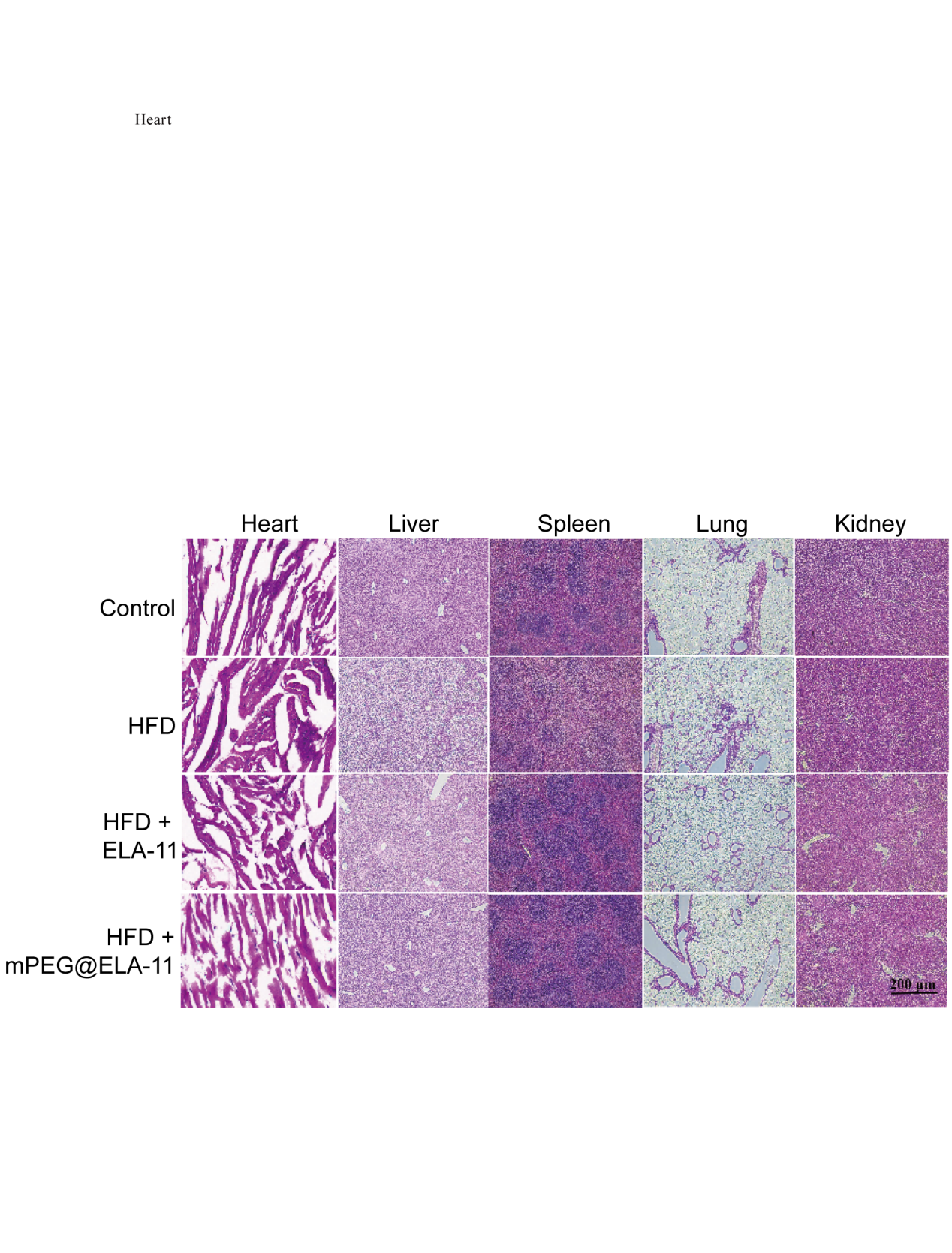
**

Histological examination of the heart, liver, spleen, lungs, and kidneys in different treatments groups which were respectively injected with the same dose (1 mg/kg) of PBS, ELA-11, and mPEG@ELA-11 via the tail vein, once every 3 days for 2 consecutive months. H&E staining showed no appreciable changes in tissue morphology. Representative images were chosen from n = 6 mice per group. Scale bar: 200 μm.
