# Supplementary figures and images for: mPEG@ELA-11 Alleviates Atherosclerosis via AKT-ER Stress-Mediated Macrophage Modulation
Source: BME Front. 2025 Nov 25;6:0203. doi: 10.34133/bmef.0203 (PMC12645589; doi:10.34133/bmef.0203)

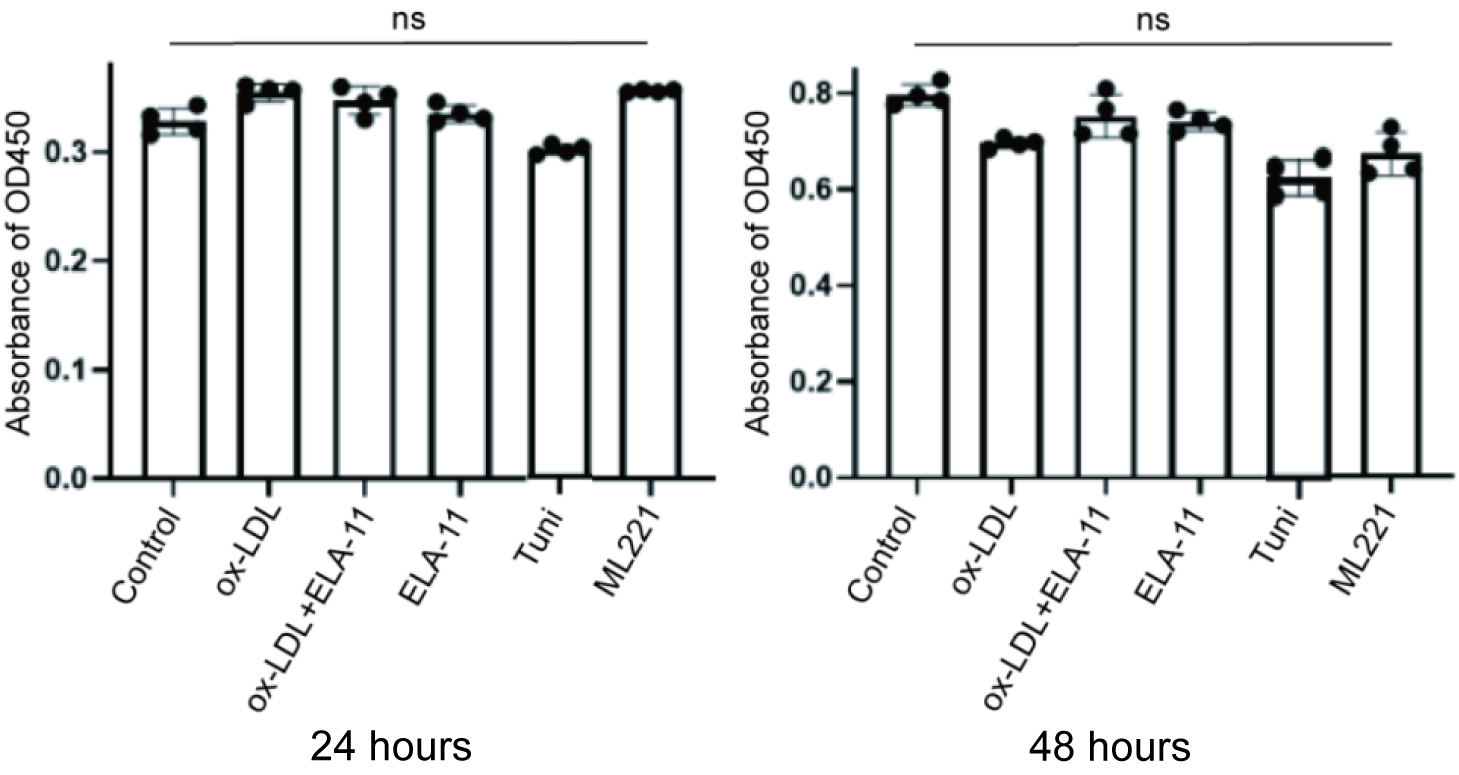

Supplement: Supplementary 1 — Figs. S1 to S3 [file bmef.0203.f1.zip › Fig S1.tif]

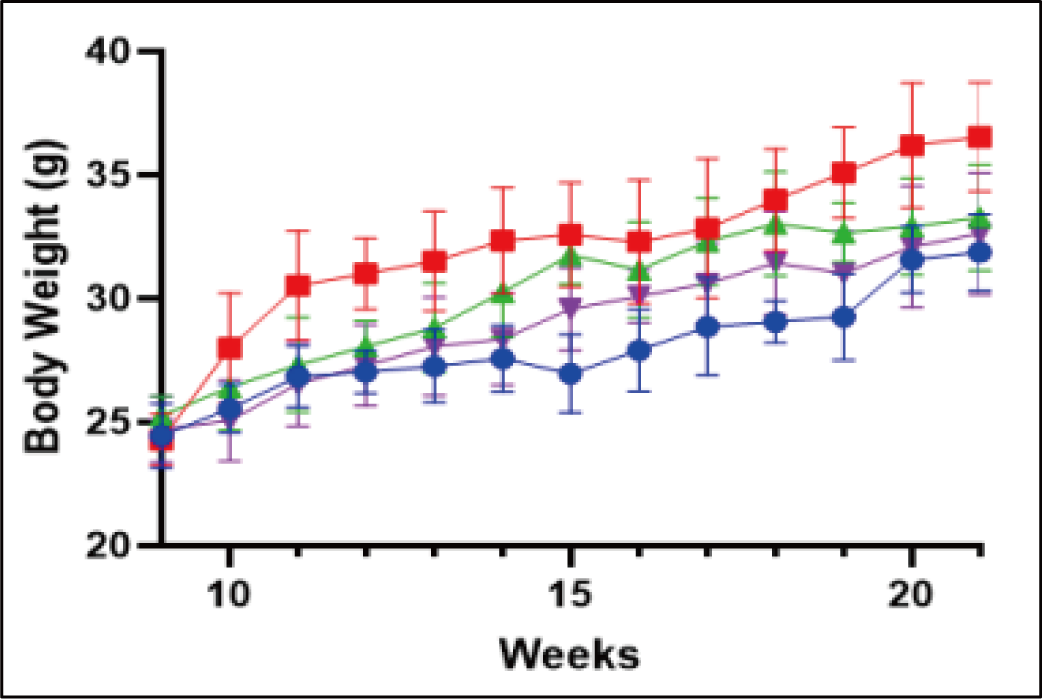

Supplement: Supplementary 1 — Figs. S1 to S3 [file bmef.0203.f1.zip › Fig S2.tif]

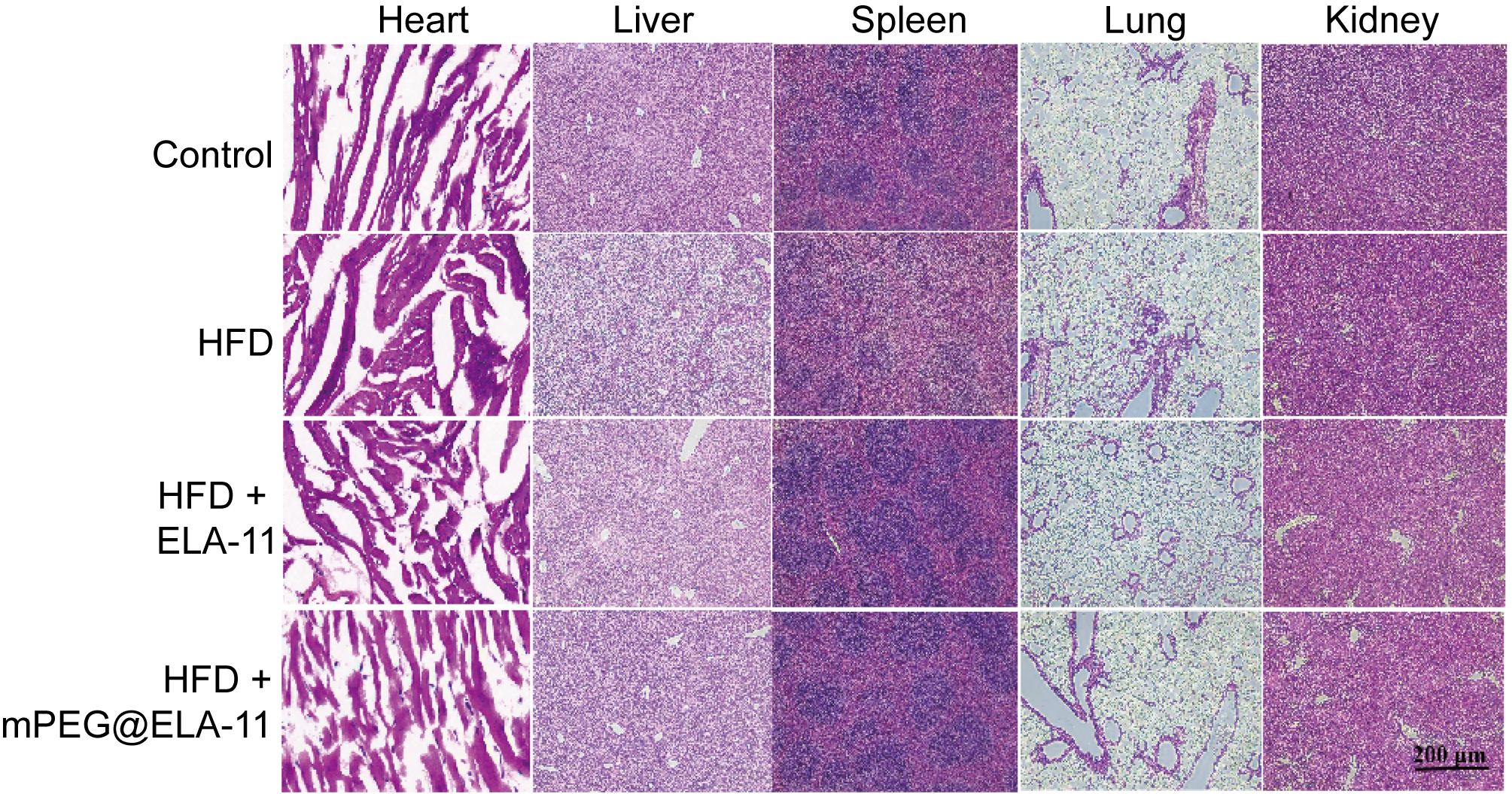

Supplement: Supplementary 1 — Figs. S1 to S3 [file bmef.0203.f1.zip › Fig S3.tif]
